# Psychometric Evaluation of the Attune & Stimulate‐Checklist for Assessing the Emotional State of People With Severe to Profound Intellectual Disabilities

**DOI:** 10.1111/jar.70170

**Published:** 2026-01-28

**Authors:** Tanja W. M. Doodeman, Carlo Schuengel, Paula S. Sterkenburg

**Affiliations:** ^1^ Faculty of Behavioural and Movement Sciences, Department of Clinical Child and Family Studies Vrije Universiteit Amsterdam Amsterdam the Netherlands; ^2^ Amsterdam Public Health Vrije Universiteit Amsterdam Amsterdam the Netherlands; ^3^ Odion Wormer the Netherlands; ^4^ Department of Assessment and Treatment Bartiméus Doorn the Netherlands

**Keywords:** arousal, construct validity, emotions, observation‐instrument, reliability, severe to profound intellectual disabilities

## Abstract

**Background:**

In response to the lack of well‐validated observation‐instruments assessing the emotions of people with severe to profound intellectual disabilities, the Attune & Stimulate‐checklist (A&S‐checklist) was psychometrically evaluated.

**Method:**

Video recordings of 102 adults with severe to profound intellectual disabilities were scored with the A&S‐checklist. Related constructs were assessed through proxy‐questionnaires and physiological measurement.

**Results:**

Good inter‐rater (ICC = 0.77–0.83) and moderate to good test–retest reliability (ICC = 0.70–0.81) were found, except for one seldomly scored A&S‐category. Moderate to strong associations were found between observed emotional states and informant reports of emotional functioning, adaptive functioning, communication and influence, and mood (*r* = 0.27–0.51; *p* < 0.05), except for negative mood. A&S‐checklist‐arousal was not statistically associated with skin conductance.

**Conclusions:**

The A&S‐checklist showed adequate psychometric properties, making assessment of emotion more feasible. The lack of a straightforward link with physiologically measured arousal invites further investigation.

**Trial Registration:** This study was pre‐registered on Open Science Framework in the project https://osf.io/pba92 (Doodeman et al. 2020, August 27). The link to this registration is: Doodeman, T. W. M., Sterkenburg, P., & Schuengel, C. (2020, August 27). The Construct and Convergent Validity of the ‘Attune & Stimulate’ checklist for parents and caregivers of persons with severe intellectual disabilities. https://doi.org/10.17605/OSF.IO/PBA92

## Lay Summary

- The Attune & Stimulate‐checklist was a reliable instrument to describe emotional states in persons with severe to profound intellectual disabilities.
- Observations with the Attune & Stimulate‐checklist were largely aligned with what informants reported about mood, emotional and adaptive functioning, and communication and influence.
- Observed arousal with the Attune & Stimulate‐checklist was not associated with skin conductance, an objective measure of arousal. The link between skin conductance and emotional wellbeing in a day‐to‐day setting may be more complex.
- The Attune & Stimulate checklist may facilitate further research on emotional wellbeing of people with severe to profound intellectual disabilities.

## 1 Introduction

The wellbeing of people with intellectual disabilities has become more of a priority over recent years, which is important especially for persons with profound intellectual disabilities, as their wellbeing may be especially low (Beadle‐Brown et al. 2009; Vos et al. 2010). However, people with severe to profound intellectual (and multiple) disabilities have no or limited ability to communicate verbally about their wellbeing. Studies into subjective wellbeing in people with severe/profound intellectual disabilities therefore need to go beyond a verbal self‐report measurement (Ware 2004). Well‐accepted and frequently used alternative methods are proxy‐questionnaires and behavioural observations, each of which has its unique limitations regarding reliability and validity in the face of often idiosyncratic signal repertoires (Maes et al. 2021). Therefore, Maes et al. recommend combining different complementary assessment methods. The current study aimed to address the need for well‐validated observation instruments measuring affect, emotions and arousal in people with severe/profound intellectual disabilities (Vos et al. 2013b).

Behavioural observations can provide insight into a person's current emotional state (Petry and Maes 2006; Vandesande et al. 2020; Vos et al. 2013b), which in this study is defined as the observed arousal (i.e., intensity of the emotion—from low to high) and valence (i.e., direction of the emotion—from negative to positive). Observational procedures focused on emotions are prone to bias, and therefore, require psychometric evaluation. Petry and Maes (2006) developed an observational method based on a multi‐perspective procedure by first drafting an individual profile of affective expressions with parents and/or professional caregivers after which the observer uses this affective profile to code video recordings. Vos et al. (2012, 2013a) used this method in a small sample in combination with a 5‐point scale ranging from ‘expressing a very negative emotion’ to ‘expressing a very positive emotion’ and validated this with physiological measures. An advantage of using an individual profile is that parents’ and caregivers’ knowledge of the participants’ idiosyncratic behaviours is used to inform coders. Disadvantages of this method are the limited predetermined categories of communicative behaviour and the lack of differentiation in subcategories, indicating that more specificity may be needed (Petry and Maes 2006). Furthermore, this procedure may be difficult to translate towards practical use.

The Attune & Stimulate‐checklist (Doodeman et al. 2018) was developed to measure the emotional state (i.e., arousal and valence) of persons with severe/profound intellectual disabilities. The observation‐instrument consists of a behavioural catalogue of 92 behavioural item‐options. The behavioural catalogue was generated by analysing behaviour of adolescents aged 10–17 with severe intellectual disabilities (Doodeman et al. 2022) and was used in an effectiveness study with 102 adults with severe/profound intellectual disabilities (Doodeman et al. 2023). This catalogue was combined with an adjusted Arousal & Valence‐scale (A&V‐scale; Sterkenburg and Frederiks 2017) to rate subtle emotional fluctuations of a person with severe/profound intellectual disabilities at any given moment. To contribute to the content validity of the Attune & Stimulate‐checklist, the researchers discussed and adjusted the proposed observation‐instrument with advisory groups consisting of parents, relatives, professional caregivers, and students. The Arousal & Valence‐scale and Attune & Stimulate‐checklist have been used as measures in experimental studies, showing promising results (Doodeman et al. 2023; Vandesande et al. 2020). Further research into the psychometric qualities is, therefore, warranted.

Measuring the emotional state of persons with severe/profound intellectual disabilities is challenging, due to their idiosyncratic and subtle expressions (Hostyn and Maes 2009). No golden standard does exist for comparison, meaning that validity can only be investigated by examining correlations with multiple related constructs. Therefore, it is important to identify measurable constructs in the nomological network of the construct *emotions* (cf. Vos et al. 2013a). In the nomological network, the construct of emotions itself contains *arousal* and *valence*. Arousal distinguishes the intensity of the emotions and valence the direction of the emotions (positive or negative). Various physiological measures are aimed at capturing arousal and/or valence (Frederiks et al. 2019; Lorang et al. 2020; Vandesande et al. 2020; Vos et al. 2012, 2013b). In addition, the construct *emotions* is closely related to the construct *mood*, with emotions being more fleeting (seconds or minutes) than mood, which may last for hours or days. Mood can be measured in people with severe/profound intellectual disabilities by the proxy‐questionnaire Mood, Interest, and Pleasure (MIPQ; Maes et al. 2015; Ross and Oliver 2003). Whereas specific emotions and moods primarily refer to transient states, people also differ in the emotions and moods that they are prone to have, reflecting individual traits. Therefore, associations are to be expected when emotional states are assessed at a given time point in a group of people who vary on trait‐like constructs relevant to mood and emotion. A related construct in the nomological network of the construct *emotions* is *quality of life*, which has a subjective component (i.e., the experience of wellbeing) and an objective component. Objective components of quality of life can be measured with the proxy‐questionnaire Quality Of Life of persons with Profound Multiple Disabilities (QOL‐PMD; Petry et al. 2007), of which the subscale *communication and influence* was most strongly associated with mood (Petry et al. 2009). This is to be expected, because communication facilitates social support for regulating emotions and addressing needs. To get to more overarching constructs in the nomological network, *emotion regulation* concerns the way people influence, manage, experience, and express their emotions (Gross 1998). Abilities contributing to adequate emotion regulation have been conceptualised as *emotional intelligence* (Mennin et al. 2007). Emotion regulation is also linked to *adaptive functioning*, in the sense that maladaptive emotion regulation is associated with psychopathology and vice versa (Aldao et al. 2010). Therefore, people with high support needs for adaptive and emotional functioning, which are measurable constructs in the current target group, have a limited ability to regulate maladaptive emotional states. To sum up, at various levels of the nomological network of the construct emotions, there are different measurable constructs in the current target group which can be linked to the emotional state measured with the Attune & Stimulate‐checklist.

The aim of the current study was to psychometrically evaluate the Attune & Stimulate‐checklist (A&S‐checklist; Doodeman et al. 2018) for the emotional state of persons with severe/profound intellectual disabilities. The first research question regarded the test–retest reliability and inter‐rater reliability of the A&S‐checklist. The second question addressed the construct validity by associating the checklist observations with (a) the related informant‐reported constructs adaptive functioning, emotional functioning, mood, and communication and influence, and (b) arousal measured based on skin conductance.

## 2 Method

### 2.1 Design

This cross‐sectional, correlational study used data from a randomised controlled trial investigating the effects of the A&S‐checklist (Doodeman et al. 2023). The sample was randomly distributed into an intervention and control group. To examine the correlation between the observed emotional states and the informant‐reported constructs, only data from the control group were used, because the emotional states might have been altered in the intervention group. No statistical differences (*p* > 0.05) were found between the groups on demographic characteristics. Data from the total sample were used for examining the reliability of the observations and the correlation with the physiological measured arousal.

### 2.2 Participants

Persons with severe to profound intellectual disabilities from nine care organisations from diverse regions in the Netherlands that provide services to persons with intellectual disabilities participated. Inclusion criteria were: (1) having a severe to profound intellectual disability (i.e., IQ below 35, estimated by a certified diagnostician); (2) being 18 years or older; and (3) living in home care facilities or attending day care facilities more than half of the week. Given the lack of a priori age ranges between which differences in the manifestation of emotions and arousal might be expected, no restrictions were set on the maximum age of the participants. Table 1 summarises the demographics of the participants for the total sample and for the control group separately.

**TABLE 1:** Demographic data of study participants.

Characteristic	Control group (*n* = 55)	Total sample (*N* = 102)
Age in years		
*M* (SD)	40.13 (15.59)	38.58 (14.53)
Range	18–81	18–81
Gender		
Female	30 (54.5%)	51 (50.0%)
Other disabilities		
Motor limitations	46 (83.6%)	82 (80.4%)^d^
Problems in visual functioning^a^	49 (89.1%)^b^	86 (84.3%)^c^
Problems in auditory functioning^a^	33 (60%)^b^	56 (54.9%)^c^
Specific health problems^a^	47 (85.5%)^c^	86 (84.3%)^d^
Behavioural and/or psychological problems^a^	34 (61.8%)^b^	63 (61.8%)^b^
Epilepsy	36 (65.5%)^b^	66 (64.7%)^c^
Diagnosis of autism	7 (12.7%)	16 (15.7%)

^a^ Based on a binary screening question.

^b^ Missing: *n* = 1.

^c^ Missing: *n* = 2.

^d^ Missing: *n* = 3.

Ethical approval was obtained from the Scientific and Ethical Review Board (VCWE) of the Faculty of Behavioural & Movement Sciences, Vrije Universiteit Amsterdam (project: “The Validity and Convergent Validity of the New Checklist ‘Attune and Stimulate’”—VCWE‐2018‐137R1). This study was pre‐registered on Open Science Framework (OSF: Doodeman et al. 2020). The study was conducted between February 2019 and July 2021.

### 2.3 Measures

#### 2.3.1 Observation of Emotion

The Attune & Stimulate‐checklist (A&S‐checklist; Doodeman et al. 2018) consists of 92 behavioural item‐options of which only the observed ones were scored on an ordinal 7‐point Arousal & Valence Likert scale, running from −3 (‘negative overstrung’) to 0 (‘neutral/low arousal’) to +3 (‘positive overstrung’). Ordinal response options within negative or positive emotional states were given to enable assessment of fluctuations in arousal. It is possible to give a behavioural item multiple scores. To illustrate, a person can laugh quietly at first and then become increasingly enthusiastic. The item ‘laughing’ is then scored as +1 and +2. The behavioural catalogue is divided into 13 categories: (1) Actions, (2) Sounds, (3) Position, (4) Whole body, (5) Upper body, (6) Lower body, (7) Arms, (8) Hands (to self, to other, to object), (9) Feet, (10) Head, (11) Eyes, (12) Nose, and (13) Mouth. Observers can add observed behaviour which is not included in the behavioural catalogue. To illustrate the application of the observation instrument, during a 10‐min video observation a coder coded 12 behavioural items of which four as ‘negative tensed’ (−2), six as ‘negative slightly tensed’ (−1), and two as ‘neutral’ (0). The coding for this hypothetical observation is illustrated in Table 2.

**TABLE 2:** Illustration of a hypothetical observation with the Attune & Stimulate‐checklist.

A&S‐scores	Number of item‐options scored	Percentage‐scores
−3. Negative overstrung	0	0
−2. Negative tense	4	33.3
−1. Negative slightly tense	6	50
0. Neutral/low arousal	2	16.7
+1. Positive slightly tense	0	0
+2. Positive tense	0	0
+3. Positive overstrung	0	0
Total	12	100

#### 2.3.2 Informant‐Reported Constructs

The Minimal Data Set (MDS) for people with intellectual disabilities, variant adults fixed‐Other (Kunseler et al. 2016) was used, supplemented with a questionnaire regarding emotional functioning.

##### 2.3.2.1 Adaptive Functioning

Available, valid^1^ scores in the participants' file on the Vineland‐Screener 0–6 (VS 0–6; Scholte et al. 2008) or the Sociale redzaamheidsschaal (SRZ; Kraijer et al. 2004) were accepted to prevent informant burden. If a valid score was not available, a family member was asked to complete the VS 0–6. The total scores of the VS 0–6 and SRZ were converted to age equivalents in months (Kraijer et al. 2004; Scholte et al. 2008).

The VS 0–6 is a Dutch adaptation of the Vineland Adaptive Behaviour Scales‐Screener (Sparrow et al. 1993). The Vineland Screener 0–6 years is a behavioural questionnaire that provides an indication of the adaptive developmental level of children aged 0–6 years and of people with intellectual disabilities with a higher calendar age but a similar developmental age (Kunseler et al. 2016). In the absence of validated and normed scales for Dutch adults with severe/profound intellectual disabilities (Ulgiati et al. 2024), the VS 0–6, which was validated for Dutch persons with intellectual disabilities aged 0–19 years old, was the best available option to get an indication of the adaptive functioning of the participants in the current sample. The VS 0–6 consists of 72 items divided into four domains: Communication skil009;years is a behavioural questionnaire that provides an indication of the adaptive developmental level of children aged 0–6 years and of people with intellectual disabilities with a higher calendar age but a similar developmental age (Kunseler et al. 2016). In the absence of validated and normed scales for Dutch adults with severe/profound intellectual disabilities (Ulgiati et al. 2024), the VS 0–6, which was validated for Dutch persons with intellectual disabilities aged 0–19 years old, was the best available option to get an indication of the adaptive functioning of the participants in the current sample. The VS 0–6 consists of 72 items divided into four domains: Communication skills, Social skills, Daily living skills, and Motor skills. The items were scored on a 3‐point scale from 0 (*person never performs this skill*) to 2 (*person usually performs this skill*). High internal consistency (Cronbach's *α* = 0.97) was found in the population of children aged 0–19 years with varying levels of intellectual and multiple disabilities, and agreement between informants was high (Scholte et al. 2008). In the general youth population, supportive evidence for content, construct, and criterion validity was found (Scholte et al. 2008). High internal consistency (Cronbach's *α* = 0.95) was also found in the current sample.

The SRZ measures adaptive behaviour, based on the Cain‐Levine Social Competency Scale (Cain et al. 1963). The SRZ consists of 31 items scored on a 4‐point scale. The items are divided in four domains: Self‐help, Communication skills, Task orientation, and Social skills. High internal consistency (Cronbach's *α* = 0.89–0.96), high test–retest reliability (*r* = 0.98), and high inter‐rater reliability (*r* = 0.95) were found in a study among both children and adults with varying levels of intellectual disabilities (Kraijer et al. 2004). The study also supported construct and criterion validity.

##### 2.3.2.2 Emotional Functioning

Available, valid^1^ scores in the participants' file on the Scale of Emotional Development‐Short (SED‐S; Morisse et al. 2017; Sappok et al. 2016) or the Scale of Emotional Development‐Revised^2^ (SED‐R2; Morisse and Došen 2016) were accepted to prevent informant burden. If a valid score was not available, the SED‐S was used.

The SED‐S is a semi‐structured interview consisting of 200 binary items (“yes” or “no”) divided in eight domains to measure the emotional functioning of persons with intellectual disabilities. The interview was conducted by the psychologist or ‘orthopedagoog'^2^ with family members and/or professional caregivers. Emotional functioning is categorised in five developmental stages: (1) Adaptation (0–6 months); (2) Socialisation (6–18 months); (3) Individuation (18–36 months); (4) Identification (3–7 years); and (5) Reality Awareness (7–12 years; Došen 2014). Studies among children and adults with mild to profound intellectual disabilities found evidence for high internal consistency (Cronbach's *α* = 0.93–0.94), construct validity (Sterkenburg et al. 2021), factorial validity (Flachsmeyer et al. 2023), and item validity, with good discriminatory power between stages on item level (Hermann et al. 2024). In the current sample, high internal consistency (Cronbach's *α* = 0.92) was found.

The SED‐R^2^ is the predecessor of the SED‐S (Sappok et al. 2016). It is more extensive than the SED‐S because it consists of 13 domains and 556 statements, from which the informants choose the most characteristic ones for the person they are involved with. High internal consistency (Cronbach's *α* = 0.95) and substantial inter‐rater reliability for total scores (ICC = 0.73) were found in persons with mild to profound intellectual disabilities (Vandevelde et al. 2016). In the current sample high internal consistency (Cronbach's *α* = 0.97) was found.

##### 2.3.2.3 Mood

Both family members and professional caregivers were asked to complete the Dutch translation of the Mood, Interest, and Pleasure Questionnaire (MIPQ; Maes et al. 2015; Ross and Oliver 2003), reporting on mood over the last 2 weeks. The MIPQ consists of 23 items divided into three subscales: Positive mood, Interest, and Negative mood. The items were scored on a 5‐point Likert scale from 0 (*never*) to 4 (*always*). High internal consistency (Cronbach's *α* ≥ 0.80), high inter‐rater (*r* ≥ 0.69), and high test–retest reliability (*r* ≥ 0.86) were found for this Dutch version of the MIPQ (Petry et al. 2010) for the scores for persons with severe/profound intellectual disabilities, as well as some evidence for the construct validity. In the current sample, high internal consistency for each of the informants (Cronbach's *α* = 0.88–0.90) and moderate inter‐rater reliability between the informants (ICC = 0.60) were found.

##### 2.3.2.4 Communication and Influence

According to the instruction manual, family members, professional caregivers, and psychologists or ‘orthopedagogen’^2^ were asked to complete the subscale “Communication and Influence” of the questionnaire Quality Of Life of persons with Profound Multiple Disabilities (CI‐QOL‐PMD; Petry et al. 2008). The 10 items of this subscale were scored on a 3‐point scale from 0 (disagree) to 2 (agree). The internal consistency of CI‐QOL‐PMD for scores for persons with profound multiple disabilities was good (Cronbach's *α* > 0.80) (Petry et al. 2009). The subscale correlated highly with the total score of the QOL‐PMD (*r* = 0.80). Evidence for the construct validity was found, including a medium correlation (*r* = 0.45) between CI‐QOL‐PMD and the MIPQ. The informant correspondence on the CI‐QOL‐PMD was medium to strong (*r* = 0.38–0.56). In the current sample, good internal consistency for each of the informants (Cronbach's *α* = 0.84–0.86) and moderate inter‐rater reliability between the informants (ICC = 0.60) were found.

#### 2.3.3 Physiological Measure of Arousal

The skin conductance sensor sock was used to measure a physiological indicator of arousal (Frederiks et al. 2019). The specially designed sensor sock and associated Android application, which is called the Flower‐App (Flower‐R3 V0.1b, Eindhoven University of Technology), were developed by the Eindhoven University of Technology, Vrije Universiteit Amsterdam, and care‐provider Bartiméus. The bio response system gives information on the activation of the sympathetic nervous system of the participant by measuring the Galvanic Skin Response in a non‐invasive and non‐interruptive manner (Frederiks et al. 2019). The system consists of a sock with two integrated fabric electrodes on the foot sole, a Shimmer 2R BioPhysical sensor (Shimmer, Dublin, Ireland), and the Flower‐App, which is connected with the Shimmer via Bluetooth. The system measures skin resistance (kΩ) in approximately 51 data points per second between the two electrodes of the sensor sock, which is converted to skin conductance by taking the inverse (1000/1 kΩ = 1 μS).

### 2.4 Procedure

The psychologists and ‘orthopedagoog’^2^ of the participating care organisations, which were certified diagnosticians, were asked to select all persons in their care who they estimated to function below the IQ threshold of 35 and meet the other inclusion criteria of the study. The coordinating researcher (first author of this paper) asked the legal guardians of these selected persons for their informed consent. The informants of each participant received the questionnaires via a digital form, or were sent by post or administered by telephone, if preferred.

Ten data collectors and 12 video coders, who were junior researchers, master's and bachelor's students of Clinical Child and Family Studies, Vrije Universiteit Amsterdam, were trained to perform the data collection and video coding according to the research protocols (i.e., protocol for data collection and protocols for video coding including consensus protocols), which was monitored by the first author of this paper. The students were allowed to code individually once they reached at least moderate inter‐rater reliability (ICC > 0.5) with the golden‐standard consensus set created by the first author of this paper and a colleague.

Participants were video recorded in a familiar setting (e.g., group home or day care facility) for 10–15 min while wearing a skin conductance sensor sock and while interacting with a professional caregiver. This was a non‐structured, free interaction. The caregiver was only asked to do the same activity for the entire moment (i.e., not: move for 5 min and lie still for 5 min), to keep the influence of movement on the signal as constant as possible. Additionally, eating and drinking activities were excluded, as metabolism can influence fluctuations in the skin conductance. No restrictions on movement were applied, which could also influence the signal. Informed consent was obtained from these professional caregivers.

Afterwards, each video was coded in two ways:

1. The video was observed and scored with the A&S‐checklist, which results in an overview of scored behavioural items per level of the 7‐point scale. The reliability parameters are presented in the Results section in Table 4. Consensus was discussed when the difference between the number of different A&V scores was two or more.
2. Using the software program The Observer XT version 15.0 (Noldus Information Technology bv., 2019), continuous coding was applied with the 7‐point Arousal & Valence‐scale. To illustrate, the coder scored the participant's level of arousal and valence while watching the video. When the level of arousal and valence changed, the coder changed the score as well. This resulted in an Arousal & Valence‐timeline per second across the video. This coding was synchronised with the measured skin conductance over time (51 data points per second), meaning that all 51 SC‐data points of 1 s were synchronised with the single observation score of that second. Good inter‐rater reliability (ICC = 0.79, *n* = 41) and test–retest reliability (ICC = 0.90, *n* = 11) were found. Consensus was discussed when in the total timeline different scores were used between the two coders (e.g., coder A used scores +1 and +2 and coder B used scores 0 and +1).

Respectively, 69% and 40% of the videos were coded independently by two different coders. At least 10% of the videos were double coded by the same coder, with 1 month apart. Using a digital randomizer, one of the two double‐coded videos was included in the analyses.

### 2.5 Data Analysis

IBM Statistical Package for Social Sciences (SPSS) version 27 was used. An alpha level smaller than 0.05 was considered statistically significant. Holm–Bonferroni corrections were applied to control the family‐wise error rate. As an amendment to the pre‐registered plan, the guidelines of Funder and Ozer (2019) were applied for interpreting effect sizes against benchmarks relevant for individual differences research. Correlations of 0.05 were defined as very small, 0.10 as small, 0.20 as medium, 0.30 as large, and 0.40 as very large.

In preparation of the analyses, percentage‐scores were calculated per scoring option of the 7‐point scale by dividing the number of scored item‐options of an A&S‐scoring option by the total number of scored item‐options times 100 (see Table 2). Then, to compare the A&S‐checklist with the overlapping constructs, four categories were distinguished in the 7‐point scale: (a) Optimal aroused behaviour (+1, +2), (b) Neutral behaviour (0), (c) Negative aroused behaviour (−3, −2, −1), and (d) Over‐stimulated behaviour (+3). The variable for negative aroused behaviour was a weighted score in which higher levels of arousal counted more than lower levels of arousal, whereas for optimal aroused behaviour both levels were equally weighted as both +1 and +2 behaviour were referred to as equally optimal.

#### 2.5.1 Reliability

The two‐way random, single intraclass correlation coefficients (ICC) were calculated over the ratio‐scores of the four A&S‐categories between two codings of the same rater (test–retest reliability) and between codings of two raters (inter‐rater reliability). Interpretation followed the guidelines of Koo and Li (2016) (poor: ICC < 0.5, moderate: 0.5 ≤ ICC < 0.75, good: 0.75 ≤ ICC < 0.9, excellent: ICC ≥ 0.90).

#### 2.5.2 Construct Validation

##### 2.5.2.1 Informant‐Reported Constructs

Optimal aroused behaviour was tested on its association with adaptive and emotional functioning, positive mood, and communication and influence. Neutral behaviour was tested for its association with interest, and negative aroused behaviour for its association with negative mood. All associations were expected to be moderately positive,^3^ except the association between neutral behaviour and interest, which was expected to be moderately negative. Over‐stimulated behaviour (+3) was not included in the analyses due to its ambiguity (it is neither positive nor negative). All associations were calculated using Pearson product–moment correlation coefficient, after inspecting the sample distributions and considering the sample size.

Mean scores were calculated from the scores of the different informants on the CI‐QOL‐PMD and the subscales of the MIPQ. Missing items on the VS 0–6, the CI‐QOL‐PMD and the MIPQ were handled according to the corresponding instruction manual. When a total score on one of the questionnaire constructs was missing, the participant was excluded from that particular analysis. The participants for whom the video was not recorded in the recall period of 2 weeks of the MIPQ (which happened frequently due to the COVID‐19 pandemic) were excluded. A sensitivity analysis was performed with the total control group sample to check robustness.

##### 2.5.2.2 Physiological Measure of Arousal

To test whether there was a monotonic increasing association between the observed arousal level with the adjusted A&V‐scale and the physiological measured arousal with the skin conductance sensor sock, the Arousal & Valence‐timeline was synchronised with the measured skin conductance (SC) over time. Then four SC‐median‐scores were calculated (μS): median SC over all SC‐data‐points while scoring neutral (0), median SC over all SC‐data‐points while scoring slightly tense (−1 and +1), median SC over all SC‐data‐points while scoring tense (−2 and +2), and median SC over all SC‐data‐points while scoring overstrung (−3 and +3) on the 7‐point‐scale. This eliminated the valence from the A&V‐scores and transformed it to only arousal‐scores from 0 to 3. By calculating the four SC‐median‐scores, it was investigated whether the level of physiologically measured arousal was higher when the observed level of arousal was higher to validate the observed level of arousal. The median is preferable to the mean and to second‐to‐second scores because it is less sensitive to extremes or measurement errors in the signal. As an amendment on the pre‐registered plan, the medians were calculated on the raw data instead of the filtered data.

Another amendment to the pre‐registered analysis plan was that multilevel modelling was used for detecting an association between the observed arousal level and the skin conductance, because multilevel models can handle incomplete data across repeated measurements (evidently, not all arousal levels were used in each participant). The step‐by‐step tutorial of Page‐Gould (2016), which is especially focused on psychophysiological data, was used to perform this analysis.

A two‐level design was used with repeated measures (level 1) nested within the study participants (level 2). The dependent variable was the SC‐median‐score (within‐subject variable, level 1). The within‐subject (level 1) independent variable was the observed arousal (0–3). The covariance structure ‘variance components’ was used in favour of the auto‐correlated structure because the order of the repeated measures is mixed up by calculating the median‐scores over all fragments scored with a certain arousal‐score. First, a baseline model (Model 1) was estimated in which no predictor was included, to assess the degree of non‐independency in SC‐data by calculating the ICC. The ICC for the baseline model was 0.94, indicating high dependency in the within‐subject data, making multilevel modelling an appropriate analysis for this dependent variable (Page‐Gould 2016). Then two other models were tested with the observed arousal level as predictor: Model 2 with random intercept and Model 3 with random intercept and slope. The significance of random effects was tested by comparing the models in a likelihood ratio test.

In this analysis, 18 participants were excluded because their observed arousal level did not vary. In addition, four participants were excluded as they participated in an alternative, online procedure during the COVID‐19 pandemic, in which it was not possible to collect physiological data. Due to movement‐induced signal disruptions, two participants were totally excluded, while six others had sections of their measurements removed.

## 3 Results

### 3.1 Reliability: Intra‐ and Interrater Reliability

Descriptive statistics for the A&S‐checklist are summarised in Table 3. This table demonstrates the range, the mean, and the standard deviation of each scorings‐option. The frequency is also displayed, which indicates the number of participants to whom the score was applied. The range indicates the percentages that occurred per A&S‐scorings‐options, for example for ‘negative tense’ a maximum of 25% is used, which means that 25% of the scored behaviours were ‘negative tensed’ and the other 75% of the behaviours were scored with other scorings‐options. The extremes were used very infrequently, especially the score ‘negative overstrung’.

**TABLE 3:** Descriptive statistics of the scoring options of the A&S‐checklist (*N* = 102).

A&S‐scorings‐options	*n* ^a^	Range	Minimum	Maximum	*M*	SD
−3. Negative overstrung	1	4.2%	0%	4.2%	0.04%	0.4
−2. Negative tense	12	25.0%	0%	25.0%	1.2%	3.9
−1. Negative slightly tense	55	42.9%	0%	42.9%	9.0%	11.1
0. Neutral/low arousal	102	90.5%	9.5%	100%	56.1%	21.5
+1. Positive slightly tense	87	71.4%	0%	71.4%	29.3%	18.8
+2. Positive tense	31	33.3%	0%	33.3%	4.0%	7.6
+3. Positive overstrung	6	14.3%	0%	14.3%	0.4%	1.9

^a^ Number of participants score is used for (*N* = 102).

The two way‐random, single intraclass correlation coefficients (ICC) examining the test–retest reliability and the inter‐rater reliability are displayed in Table 4. The inter‐rater reliability was good for each A&S‐category, except for over‐stimulated behaviour, which may be explained by the low occurrence of this score in the sample. The test–retest reliability was moderate to good, again with the exception of the category over‐stimulated behaviour, which was scored too scarcely to calculate an ICC for.

**TABLE 4:** The two‐way random ICCs per A&S‐category.

A&S‐category	Inter‐ICC^a^	Intra‐ICC^b^
Negative aroused behaviour (−3, −2, −1)	0.83	0.74
Neutral behaviour (0)	0.77	0.70
Optimal aroused behaviour (+1, +2)	0.83	0.81
Over‐stimulated behaviour (+3)	0.39	0.00

^a^ *n* = 70, *k* = 2.

^b^ *n* = 15, *k* = 2.

### 3.2 Construct Validity

#### 3.2.1 Informant‐Reported Constructs

Table 5 presents the descriptive statistics of the six constructs: emotional functioning, adaptive functioning, communication and influence, positive mood, interest, and negative mood. Table 6 shows the correlations between the outcome variables measured with the A&S‐checklist (optimal aroused, negative aroused, and neutral behaviour) and the related constructs.

**TABLE 5:** Descriptive statistics of the related constructs.

Related constructs	*n* ^a^	Range	Minimum	Maximum	*M*	SD
Emotional functioning (phase 1–5)	36	2	1	3	1.3	0.5
Adaptive functioning (months)	50	49	2	51	12.4	9.9
Communication and influence (%)	55	71.2	13.8	85.0	49.9	15.7
Positive mood (score 0–36)	22^b^	24	3	27	15.1	7.5
Interest (score 0–28)	22^b^	17	3	20	9.8	5.3
Negative mood (score 0–28)	21^b^	10	18	28	22.4	2.6

^a^ In these analyses only the data of the in Doodeman et al. (2023) described control group (*n* = 55) were used (see Section 2.1).

^b^ Only the questionnaires completed in the recall period of 2 weeks were used.

**TABLE 6:** Associations between the categories of the A&S‐checklist and related constructs.

Related constructs	*n*	Optimal	Neutral	Negative	95% Lower bound^a^	*p* ^b^
Emotional functioning	36	0.49			0.24	< 0.01
Adaptive functioning	50	0.28			0.04	< 0.05^c^
Communication & influence	55	0.30			0.09	< 0.05^c^
Positive mood	22	0.51			0.19	< 0.01
Interest	22		−0.30		0.06^a^	0.09
Negative mood	21			0.16	−0.23	0.25

^a^ For the correlation with the construct Interest, the 95% upper bound is given.

^b^ One‐tailed.

^c^ *p*‐Value was not smaller than the adjusted rejection criteria according to the Holm–Bonferroni correction.

The positive correlations between optimal aroused behaviour and the constructs positive mood and emotional functioning exceeded the benchmark expectations. This also applied to the strong, positive correlation between optimal aroused behaviour and communication and influence. The strength of the correlation between optimal aroused behaviour and adaptive functioning of the participant was, as expected, moderately positive. The association between neutral behaviour and interest was, as expected, negative, but not statistically significant, even as the positive correlation between negative aroused behaviour and negative mood.

As shown in the table, the sample sizes of the analyses with the data of the MIPQ were substantially lower than for the other analyses, due to the missing values caused by informants answering the questionnaire outside the response period of 2 weeks from the video recording (see Section 2 for more information). To check the robustness of the results, the correlations were also calculated for the complete sample of 55 participants (i.e., sensitivity analyses). This analysis showed robustness in the sense that the very large, positive correlation became a large positive correlation (positive mood; *r* = 0.36, *p* < 0.01, 95% CI‐LB = 0.14) and the large, positive correlation became moderately positive (interest; *r* = −0.27, *p* < 0.05, 95% CI‐UB = −0.05), of which the last mentioned *p‐*value was not statistically significant after applying the Holm–Bonferroni correction. The small, positive correlation became too small (negative mood; *r* = 0.04, *p* = 0.39, 95% CI‐LB = −0.19).

#### 3.2.2 Physiological Measure of Arousal

Table 7 shows the means and standard deviations of the medians of the skin conductance (SC; μS) per level of observed arousal (*n* = 78). Descriptively, there was a monotonic increase from SC‐median by an arousal score of 0 to SC‐median by an arousal score of 2. The SC‐median by an arousal score of 3 was the lowest, while it was expected to be the highest. Only in four participants, an arousal score of 3 was used (see Table 7).

**TABLE 7:** Descriptive statistics of the medians of the SC per level of observed arousal (*n* = 78).

SC‐medians per level of arousal^a^	*n*	*M* ^b^	SD^b^	Minimum^b^	Maximum^b^
Median SC Arousal 0 (μS)	66	7.8	19.8	0.2	117.7
Median SC Arousal 1 (μS)	78	9.2	23.8	0.2	135.1
Median SC Arousal 2 (μS)	31	10.9	30.8	0.2	163.9
Median SC Arousal 3 (μS)	4	6.9	5.4	0.2	12.7

^a^ The SC‐median‐scores were calculated as follows: median SC while scoring neutral (0), slightly tense (−1 and +1), tense (−2 and +2), and overstrung (−3 and +3) on the 7‐point‐scale.

^b^ × 10^−4^.

Table 8 shows, contrary to expectations, that in neither model did the observed arousal score predict skin conductance. However, including the observed arousal level as a predictor with a random intercept and random slope (Model 3) did improve the model fit significantly, *X* ^2^(2) = 21.48, *p* < 0.001, suggesting that the random effect (especially the slope) explained a significant portion of the variance. To check the robustness of the results, the analysis was repeated only for the arousal level 0 to 2, as an observed arousal level of 3 only occurred in four participants and seems not to fit into the linear relationship of a monotonic increase of skin conductance according to the observed arousal levels. This analysis made no change to the results, which implies robustness of the results.

**TABLE 8:** The association between the observed arousal and the SC reactivity.

Fixed effects	*B* ^a^	SE^a^	*p*	*X* ^2^
Model 1				—
Intercept	9.65	2.69	< 0.001	
Model 2				0.25
Intercept	9.62	2.81	< 0.001	
Arousal	0.05	0.71	0.950	
Model 3				21.48*
Intercept	8.95	2.41	< 0.001	
Arousal	0.19	0.99	0.846	

*Note:* Model 2 includes only a random intercept. Model 3 includes a random intercept and slope.

^a^ × 10^−4^.

* *p* < 0.001.

## 4 Discussion

Study findings supported good inter‐rater and moderate to good test–retest reliability of emotional states assessed with the Attune & Stimulate‐checklist. Construct validity was broadly supported by associations with adaptive and emotional functioning, communication and influence, and mood in the expected directions. However, no supportive evidence was found for the validity of the category negative aroused behaviour and weak evidence for the category neutral behaviour. No statistically significant association was found between observed arousal with the A&S‐checklist and physiologically measured arousal.

The generally good reliability in this study is a positive outcome, as the reliability in observational studies appears to be a point of attention in the current target group (Maes et al. 2021). In the current study, to ensure good inter‐rater reliability, coders were trained in the use of a scoring manual. Although recommended by Maes et al. (2021), no personal profiles were used and parents or professionals were not involved in the coding procedure. This suggests that reliable coding is possible without this extra effort, which is promising for studies with large sample sizes for which this might be too time consuming. Only the reliability of the category over‐stimulated behaviour was insufficient, which may be explained by the low occurrence of this score in our study. This score was only used for six participants, which is positive given the setting in which the videos were recorded, which was a daily interaction between the participant and a professional caregiver. As it is an open question whether there are settings or situations in which overstimulated behaviours occur more frequently, a larger sample in a similar setting is needed to investigate the reliability on this category.

Regarding the construct validity, the validation of the category optimal aroused behaviour was the most robust, compared to finding weak evidence for the category neutral behaviour and no evidence for the category negative aroused behaviour. The category of optimal aroused behaviour was associated with four theoretically related constructs. The correlation with positive mood was the highest, as expected, as this construct has the strongest connection with optimal aroused behaviour within the nomological network. Furthermore, the higher the emotional and adaptive functioning and the possibilities to communicate and have influence, the higher the optimal aroused behaviour, which was expected as more skills are developed to regulate emotions or possibilities are available to express them. The negative correlation between neutral behaviour and the construct interest was not statistically significant in the small sample, but it was in the bigger sample. This indicates that there is some supportive evidence for the validity of this category. The non‐significant small correlation between the category negative aroused behaviour and negative mood may be explained by the low occurrence of high negative arousal scores. In conclusion, the validity of the category optimal aroused behaviour was the most robust in the current study.

The observed level of arousal was not associated with skin conductance. Frederiks (2021) also found low agreement between behavioural observations and skin conductance. However, in the study of Frederiks (2021), the reliability and validity of the behavioural observations were a point of discussion, this explanation is less plausible for the current study, given the psychometric support for the A&S‐checklist. Although a descriptive monotonic increase was found in skin conductance from observed arousal level 0 to 2, a drop in skin conductance at observed arousal level 3 occurred, even below the skin conductance at observed arousal level 0. This was not expected and reduced any association. This finding is reminiscent of the phenomenon called hypo‐arousal. Hypo‐arousal is thought of as a shift from a state of rapidly escalating hyperarousal. Hypo‐arousal refers to a state of reduced physiological arousal and its outward manifestation in freezing or stilling behaviour may lead to appearance as absent, lethargic, or drowsy (Porges 2003). However, as the interactions were daily caregiving situations, this extreme dynamic was not expected and, additionally, if these high levels of arousal (ending up in hypo‐arousal) occurred in the study, more −2 and −3 scores were expected. In the current study, the number of participants scoring an arousal score of 3 was too low to draw any conclusions from this finding. The statistically significant random slope effect for the association between observed arousal and skin conductance indicates the need for more complex models to understand the connection between this physiological signal and observable behaviour.

### 4.1 Limitations

For validating an instrument specifically developed for the group of persons with severe/profound intellectual disabilities, the researchers had to deal with well‐known limitations of related measures. Measuring subjective themes, such as emotions and mood, is known to be challenging in persons with severe/profound intellectual disabilities, as each source of information (proxy‐questionnaires, observations, physiological measures) has its own limitations (Maes et al. 2021). First, the validity of proxy‐questionnaires is questioned often, because it is based on interpretations that others make of what a person experiences, knowing that interpreting emotions of people with severe/profound intellectual disabilities is challenging partly due to their idiosyncratic expressions (Hostyn and Maes 2009). Therefore, multiple informants were involved in the current study, as recommended by Maes et al. (2021). Another problem is the lack of specifically developed and evaluated instruments for the current target group, because frequently instruments from other target groups were used, of which the suitability is not guaranteed (Maes et al. 2021). These instruments, for instance, might be insufficient to capture subtlety in behaviour (Vandesande et al. 2019). This is for instance the case with the SED‐R/S, which is limited in the distinctiveness for the current target group (Sterkenburg et al. 2021; Van Keer et al. 2022). However, two proxy‐questionnaires were used which were especially developed for the current target group (QOL‐PMD and MIPQ). Second, although there are some promising results in studies using physiological measurement (Frederiks 2021; Lima et al. 2013; Lorang et al. 2020; Munde et al. 2012; Vandesande et al. 2020; Vos et al. 2012; Vos et al. 2013b), research in this area is not yet conclusive regarding the validity and interpretation of this measurement. To deal with the limitations of each of the sources, different methods of assessment were combined, as recommended by Maes et al. (2021).

A limitation of the current study was that only the control group was used for most of the analyses, which resulted in a lower sample size than desired. In this sample size the occurrence of stress (−3, −2, +3, according to Doodeman et al. 2022) was low. This may have influenced the psychometric evaluation of the categories negative aroused behaviour and overstimulated behaviour. Additionally, the A&S‐category of overstimulated behaviour was not included in the validation and the categories neutral behaviour and negative aroused behaviour were only associated with one related construct in a small sample. Future research should focus on a more robust validation process for those three categories, in a larger sample with a higher occurrence of stress.

### 4.2 Implications and Recommendations

In the current study, evidence was found for the reliability and validity of the A&S‐checklist for use in research to the emotional wellbeing of persons with severe/profound intellectual disabilities in day‐to‐day situations. This may indicate that this observation‐instrument is suitable to use in future research in the current target group, for instance for the evaluation of interventions. Previous effect research has shown that the category optimal aroused behaviour in particular can demonstrate an effect on the emotional wellbeing of people with a severe/profound intellectual disability (Doodeman et al. 2023). The psychometric evaluation of the other categories of the A&S‐checklist could benefit from supplemental findings in a larger sample. Additionally, future research will have to investigate to what extent the A&S‐checklist discriminates hypo‐arousal from neutral/low‐aroused behaviour, to prevent any misinterpretations.

Our study findings suggest that there is no unequivocal association between behavioural observations of arousal and physiological measure of arousal in persons with severe/profound intellectual disabilities. Physiological measures such as skin conductance, but also heart rate (Kildal et al. 2021), might not provide one‐on‐one insight into internal mental states such as emotions, arousal, or pain, but require consideration of its dynamics and context. Additionally, more insight is needed in the complex interpretation of skin conductance and individual differences before such data can elucidate emotional wellbeing in day‐to‐day situations. In contrast to studies in day‐to‐day situations where small, unpredictable changes need to be measured, studies in experimental paradigms using stressors that significantly alter arousal have demonstrated the added value of using physiological measures (Vandesande et al. 2020). However, for the use of physiological measures for studying daily fluctuations further development is needed especially focussing on influencing factors (e.g., metabolism, movement) and adequate interpretation of the physiological measures. Although the measurement instrument (i.e., the skin conductance sensor sock; Frederiks 2021) is suitable to the current target group, more knowledge is needed on the use of physiological measurement in day‐to‐day measures to be of added value in addition to behavioural observations.

Moreover, this study found evidence for good psychometric quality of elements of a newly developed observation instrument. Observation is a commonly used method of measurement in researching the current target group, but is also frequently discussed because of the difficulties in interpreting behavioural observations, especially in subjective themes such as emotions (Maes et al. 2021). This study indicates that the A&S‐checklist can be used for behavioural observation in the current target group.

## 5 Conclusion

In the current study an observation‐instrument especially developed for the current target group was psychometrically evaluated. Emotional state was overall reliably assessed with the Attune & Stimulate‐checklist. Especially the category optimal aroused behaviour was largely supported as a valid indicator of emotional state, but also some evidence was found for the category neutral behaviour. Future research could focus on the psychometric evaluation of the instrument in a larger sample with a higher occurrence of stress. The observations of arousal with the Attune & Stimulate‐checklist were not associated with a physiological measure of arousal, suggesting that more complex interpretive models need to be developed to be able to use physiological measures in research to capture fluctuations in emotional wellbeing in a day‐to‐day setting. However, the Attune & Stimulate‐checklist appears to be a promising measure for the emotional state of persons with severe to profound intellectual disabilities, providing a viable alternative to informant report. The research field could benefit from this well‐validated observation‐instrument for the category optimal aroused behaviour, especially developed for the current target group.

## Funding

This work was supported by ZonMw (The Netherlands Organization for Health Research and Development) located in the Hague (The Netherlands), under Grant 845004001 (the programme of “Gewoon Bijzonder”) and by Bartiméus Fund (Grant nr P00748).

## Ethics Statement

Ethical approval was obtained from the Scientific and Ethical Review Board (VCWE) of the Faculty of Behavioural and Movement Sciences, VU Amsterdam (project: “The Validity and Convergent Validity of the New Checklist ‘Attune and Stimulate’”—VCWE‐2018‐137R1).

## Consent

Informed consent was asked from the legal guardians of the persons with severe to profound intellectual disabilities meeting the inclusion criteria of the study.

## Conflicts of Interest

The authors declare no conflicts of interest.

## Data Availability

The data that support the findings of this study are available from the corresponding author upon reasonable request.

## Endnotes

^1^
To verify the validity of the scores, the psychologist or ‘orthopedagoog’ involved in the participant's care was asked to assess whether the score remained appropriate based on the participant's current level of functioning.

^2^
‘Orthopedagoog’ is a Dutch term referring to a specialised, licensed educationalist offering guidance and treatment in deviant, problematic parenting and development situations, for example in case of a developmental or learning delay or an impairment. ‘Orthopedagogen’ have an academic degree in “Orthopedagogiek” and are scientifically and practically trained diagnosticians and interventionists in the pedagogical relationship between the client and his environment. They therefore provide educational advice to educators (parents or professionals) and other specialists within healthcare.

^3^
The researcher did not recode the scores on the subscale negative mood as instructed in the manual to ensure that high scores represent high negative mood.
